# Monoclonal Antibodies to Thyrotropin Receptor With Thyroid‐Stimulating Activity Activate the NF‐κB Pathway to Induce Chemokine Expression

**DOI:** 10.1111/jcmm.70647

**Published:** 2025-06-11

**Authors:** Yang Yang, Chen Hui

**Affiliations:** ^1^ Department of Endocrine The second hospital & clinical medical school,Lanzhou university Lanzhou Gansu China; ^2^ The Second Clinical Medical College Lanzhou University Lanzhou Gansu China

**Keywords:** anti‐TSHR mAbs, chemokine, Graves' disease, NF‐κB

## Abstract

The A subunit of thyrotropin receptor (TSHR) is thought to be the crucial gene mediating stimulatory autoantibodies in Graves' diease (GD), but it remains unclear what the molecular basis of this pathological antibody response is. Stimulatory TSHR autoantibodies may induce activation of multiple signalling pathways in GD, modulate chemokine exposure and further stimulate immune imbalance. In this study, we prepared TSHR 289 protein by using insect baculovirus expression, adenovirus‐expressed TSHR289 immunised mice, and obtained three mouse anti‐TSHR monoclonal antibodies (mAbs), 1A4, 7C3 and 22B1, by the hybridoma technique. Flow assay and ELISA tests tested the activity and competitive binding of the mAbs. After mAbs stimulation of human thyrocytes, RT‐qPCR and ELISA were used to detect the expression of chemokine; Western blotting detected the expression of CCL19 and the level of phosphorylation of NF‐κB. Nanogram concentrations of the IgG mAbs 1A4, 7C3 and 22B1 and their Fab induce TSHR stimulation. TRAb in the serum of GD patients competitively inhibits the binding of HRP‐conjugated mAbs to TSHR on the coated plate. Injection of micrograms of 7C3 resulted in elevated serum thyroxine and columnar and papillary hyperplasia of thyroid follicular epithelial cells. All three mAbs induced distinct expression of CCL2, CCL19 and CCL5 by activating canonical and non‐canonical NF‐κB signalling pathways in human thyrocytes. Collectively, we obtained three mouse anti‐TSHR mAbs which provide an improved approach to characterise the molecular basis of this pathological response, and confirmed that stimulating antibodies activate NF‐κB, inducing chemokines involved in the autoimmune response.

## 1 Introduction

Graves' disease (GD) is a common autoimmune disorder mediated by thyrotropin receptor (TSHR) autoantibodies [1]. Stimulating TSHR autoantibodies (TRAb) are characterised by competition with TSH for binding TSHR and activating TSHR, resulting in overstimulation of the thyroid gland and hyperthyroidism. TSHR is a G protein‐coupled receptor expressed mainly on the plasma membrane of thyrocytes, with a large extracellular domain, seven transmembrane regions and a short cytoplasmic tail [2]. After posttranslational processing, TSHR cleaves into two subunits linked by disulfide bonds: the ligand‐binding extracellular region A subunit and the transmembrane B subunit [3]. A portion of the A subunit may be shed from the cell surface to be presented to T cells [4], inducing a humoral stimulated TSHR autoantibody response; therefore, the TSHR A subunit is considered to be the primary immunogen for GD [5, 6].

Activation of multiple signalling pathways including canonical nuclear factor κB (NF‐κB) by TSHR autoantibodies has been reported previously [7, 8]. NF‐κB‐activating agents can induce the phosphorylation of Iκκ's, targeting them for rapid degradation through a ubiquitin‐proteasome pathway, releasing NF‐κB to enter the nucleus, where it regulates gene expression. It has been suggested that NF‐κB is able to activate the promoter regions of many chemokines [9]. Chemokines have been found to be involved in inflammatory processes and immune regulation of multiple diseases [10, 11], including autoimmune thyroiditis [12, 13]. Previous studies have found that CCL2 and CCL5 may be involved in the progression of GD [14, 15], of which CCL5 has been found to be closely related to TRAb [16]. It is still unclear whether TSHR autoantibodies can induce the expression of chemokines. Whether autoantibodies against TSHR can activate chemokines by inducing NF‐κB remains to be confirmed.

In this report, we obtained three mAbs with different activities against TSHR by targeting the TSHR A subunit as the main immunogen, and we confirmed their different properties by in vivo and in vitro experiments, verifying that they regulate the differential expression of chemokines through the activation of canonical and non‐canonical NF‐κB pathways.

## 2 Materials and Methods

### 2.1 Materials and Experimental Instruments

Sf9 cells and High Five cells were obtained from Thermo Fisher Scientific, which were grown in Sf‐900 II SFM medium (10902104, Thermo Fisher Scientific, Waltham, MA, USA) and SIM HF Expression Medium (MHF1, Sino Biological Inc., Beijing, China). SP2/0 mouse myeloma cells and CHO‐k1 cells were obtained from Lanzhou Institute of Biological Products Co. Ltd. (Lanzhou, China). The Nthy‐ori 3‐1 cell line was provided by the Sun Yat‐Sen University Cancer Center (Guangzhou, China). SP2/0 mouse myeloma cells and the Nthy‐ori 3‐1 cell line were cultured in RPMI‐1640 medium (31800089, Thermo Fisher Scientific) plus 10% fetal bovine serum (FBS) (10099141C, Thermo Fisher Scientific) and 1% penicillin/streptomycin (15070063, Thermo Fisher Scientific). CHO‐k1 cells were maintained in F12K medium (21127022, Thermo Fisher Scientific) containing 10% FBS and 1% penicillin/streptomycin. Primers were synthesised by Genscript Biotech (Nanjing, China). Polyethylene Glycol (PEG) 1450 (C7079, Bioss, Beijing, China), Hypoxanthine‐Aminopterin‐Thymidine (HAT) Supplement (100×) (21060017, Thermo Fisher Scientific), Hypoxanthine and Thymidine (HT) Supplement (50×) (11067030, Thermo Fisher Scientific), TSHR antibody (3B12, sc‐53542, Santa Cruz Biotechnology Inc., Dallas, USA) for western blot, rabbit anti‐mouse IgG‐horseradish peroxidase (HRP) (A9044, Merck, Darmstadt, Germany) and HRP Conjugation Kit (ab102890, Abcam, Cambridge, UK) were used.

### 2.2 Expression of TSHR289 in Insect Cells

A TSHR289 construct was amplified using the first 289 amino acid residues of human TSHR as a template which added a BamHI restriction site to the N terminus and also a six‐histidine(6 × HIS) tag, stop codon, and an EcoRI restriction site to the C terminus. The amplified product was cloned into pFastBac1, which also constructed a recombinant empty plasmid containing green fluorescent protein (GFP) (Sangon Biothech Co. Ltd., Shanghai, China). The recombinant plasmid containing the target gene was identified by double digestion and sequencing. The cDNA was used to generate recombinant protein in the Bac‐to‐Bac baculovirus expression system using the virus signal peptide in place of the TSHR signal peptide (amino acid residues 1–21). The recombinant plasmid amplified in DH5a competent cells was confirmed by nucleotide sequencing and used to transform DH10Bac cells. Recombinant Bacmid DNA was transfected into Sf9 cells to obtain and amplify recombinant baculovirus stock. Viruses were used to infect High Five cells, and GFP‐containing recombinant empty plasmid viruses infected cells simultaneously to observe infection efficiency. We inoculated the virus at an MOI ratio of 5 pfu/cell and observed optimal protein expression 3 days post‐infection. Then, the cell culture supernatant was centrifuged, and 4%–15% Precast‐Gel Tris‐Glycine PAGE (C651104, Sangon Biothech Co. Ltd., Shanghai, China) was used to determine the protein expression level and expression form. After deglycosylation, nickel column affinity chromatography was used for the purification of the TSHR289 recombinant protein. SDS‐PAGE and Western blot were used to detect the purity and nature of the proteins, respectively.

### 2.3 Immunisation of Animals for Hybridomas

Six‐week‐old female BALB/c mice (Lanzhou Veterinary Research Institute, Chinese Academy of Agricultural Sciences) were raised in a specific pathogen‐free (SPF) environment of animal care facilities at Lanzhou University Second Hospital (Lanzhou, China). Animals were kept under standard housing conditions (23°C ± 2°C, 55% ± 10% RH) in groups of five animals in individual ventilated cages, with free drinking water and standard food (20% calorie protein, 10% calorie fat and 70% kcal carbohydrates). All animal care procedures and treatments were performed according to the Guide for the Care and Use of Laboratory Animals and were approved by the local authorities at Lanzhou University Second Hospital, China (approval number D2023‐026).

Eighteen mice received 10^8^ plaque‐forming units (pfu) of adenovirus carrying the TSHR 289 gene (Ad‐TSHR289) (construction of recombinant adenovirus by Genechem, Shanghai, China), and nine mice received the same dose of an adenovirus expressing green fluorescent protein (Ad‐EGFP) as controls. Mice in the respective groups were injected in the left and right quadriceps with 25 μL phosphate‐buffered saline (PBS) containing Ad‐TSHR289 three times at 3‐week intervals. The animals were bled 1 week after the second injection and subsequently sampled again 1 week after the third injection and tested individually for binding activity to recombinant TSHR289 protein. Animals with the highest serum antibody titers received a third injection of Ad‐TSHR289. One week later, mice were injected in the tail vein with 50 μg of purified TSHR 289 proteins. The animals were sacrificed 3 days later, and the spleens were removed aseptically for hybridoma production followed by the collection of blood by cardiac puncture for serum.

### 2.4 Preparation of Mouse Anti‐TSHR289 mAbs

Mouse splenocytes were fused to SP‐2/0 cells using 50%PEG for hybridoma production in RPMI 1640 medium containing 20% FBS and 1% penicillin‐streptomycin and plated into 96‐well plates. Hybridomas were selected under HAT medium and HT medium. Approximately 11 days after fusion, culture supernatants were screened by enzyme‐linked immunosorbent assay (ELISA) for antibodies to TSHR289. ELISA plates (446469, Thermo Fisher Scientific) were coated with purified TSHR289 protein, and detection was performed with rabbit anti‐mouse IgG HRP. Hybridomas that were positive were recloned three times by limiting dilution to obtain monoclonal cell lines. Monoclonal cells were expanded in culture, cell supernatants were harvested by centrifugation, injected intraperitoneally into 8‐week‐old female BALB/c mice, and ascites were collected at day 7–10, salinized by ammonium sulphate precipitation, resuspended by centrifugation and purified by Protein G, purity assessed by SDS‐PAGE. Thereby obtaining three mouse mAbs for the TSHR289 with high purity. The mAbs were isotyped using a mouse monoclonal isotyping kit (11493027001, Merck, Darmstadt, Germany). HRP conjugation of anti‐TSHR289 mAbs was performed using an HRP conjugation kit.

### 2.5 Surface Plasmon Resonance Analyses and Detection

The kinetic properties that determine the affinity of anti‐TSHR289 mAbs and purified TSHR 289 proteins were determined by surface plasmon resonance (SPR) on a Biacore T200 (GE Healthcare, Chicago, IL, USA) using Series S carboxymethylated dextran sensor chips (CM5) sensor chips (GE Healthcare Bio‐Sciences AB, Uppsala, Sweden). HBS‐EP was used as a running buffer. A standard amine coupling procedure was applied to immobilise purified TSHR 289 proteins onto the CM5 surface. Briefly, purified TSHR289 protein (pH 4.5, 10 mM sodium acetate diluted to a concentration of 20 μg/mL) was captured onto EDC/NHS‐activated CM5. Around 890 response units (RU) of captured protein were immobilised in each channel. Three different antibodies were diluted to 1.6 μg/mL in HBS‐EP and captured by the purified TSHR 289 proteins surface, in individual flow cells, at a flow rate of 30 μL/min. Following this, the surface was regenerated using 1 or 2 pulses of 1.5 M Gly‐HCl, depending on the extent of binding. All experiments were performed at 25°C. Kinetic parameters were determined using Biacore T200 Evaluation Software V3.0.

### 2.6 Determination of the Binding Capability of Anti‐TSHR289 mAbs With CHO‐k1‐TSHR

Lentivirus (LV) containing the TSHR holoreceptor (1‐764aa) sequence with EGFP and empty vector control (Ctr‐EGFP; non‐targeting) was purchased from Shanghai Genechem Co. Ltd. CHO‐k1 were seeded into six‐well plates and cultured to 70%–80% confluence. Subsequently, lentiviral transduction was performed using HiTransG P virus infection reagent according to the manufacturer's instructions. Following 72 h of transfection, puromycin was added to the cell culture medium to screen for CHO‐k1‐TSHR recombinant cells that stably expressed TSHR at the cell membrane. Finally, the fluorescence expansion intensity of CHO‐k1‐TSHR cells was detected by inverted fluorescence microscopy (Axio Vert.A1, ZEISS, Oberkochen, Germany) and flow cytometry (Accuri C6, BD, New Jersey, USA). The expression levels of TSHR were detected by RT‐qPCR and Western blotting.

Stable CHO‐k1‐TSHR recombinant cells were harvested from a T25 cell culture flask using PBS, with CHO‐k1 cells and CHO‐k1‐Ctr‐EGFP used as control cells. After washing twice with PBS containing 2% Bovine albumin (BSA) (A600332, Sangon Biotech Co. Ltd., Shanghai, China), the cells were incubated for 30 min at room temperature in 100 μL of the same buffer containing 10 μg anti‐TSHR289 mAbs. After rinsing, the cells were incubated for 45 min with 100 μL PerCP‐conjugated goat anti‐mouse IgG (1:100) (405314, Biolegend, San Diego, CA, USA), washed and analysed using a BD Accuri C6 flow cytometer. To detect the binding capability of the Fab segment of the antibodies, we blocked the non–antigen‐specific binding of the TSHR antigen to the FC segment receptor using antibody (553142, BD, New Jersey, USA) that specifically recognises the mouse FC segment, all procedures according to the reagent vendor's instructions.

### 2.7 Measurement of mAbs Thyroid‐Stimulating Activities

Stable CHO‐k1‐TSHR recombinant cells were seeded into 96‐well plates (50,000 cells per well) and incubated for 24 h in F12k complete medium. The next day, F12k complete medium was removed and cells were domesticated in serum‐free medium for 2 h. Purified mAbs IgG and Fab diluted in F12k supplemented with 1% BSA and 0.5 mM 3‐isobutyl‐1‐methylxanthine (I5879, Merck, Darmstadt, Germany) were added to each well in triplicate and incubated for 4 h at 37°C. After washing three times with PBS, 150 μL cell lysate was added to each well, frozen and thawed three times repeatedly at −80°C and finally overnight at −80°C. The next day, samples were assayed for cAMP using a competitive immunoassay ELISA according to the manufacturer's protocol (SKGE002B, R&D Systems, Minnesota, USA). The results were expressed as picomoles per millilitre.

### 2.8 Competitive ELISA for Anti‐TSHR289 mAbs Characterisation

A total of 46 human sera including 32 TRAb positive and 14 TRAb negative serum samples were used in this study. The study was approved by the institutional ethics committees (approval number 2023A‐002). TRAb‐positive serum from patients with initial clinical diagnosis of GD without any treatment, and negative serum from healthy individuals without autoimmune disease or any other disease. For the competitive binding assays, competitive ELISA was designed to analyse the capability of TRAb in sera from GD patients to inhibit the binding of anti‐TSHR289 mAbs to TSHR protein. First, direct ELISA was used to evaluate the binding of different dilutions of HRP‐conjugated anti‐TSHR 289 mAbs to TSHR coated on ELISA plates (TRE/96/2A, RSR Limited, Cardiff, UK). Subsequently, for the competitive ELISA, human serum was thoroughly mixed 1:1 with HRP‐conjugated mAbs at the optimal dilution, added to the ELISA plate coated with TSHR and incubated for 60 min at 37°C. After incubation, the plate was washed again and incubated with freshly prepared 3,3′‐5,5′‐tetramethyl benzidine (TMB) peroxidase substrate for 15 min at 37°C. The reaction was stopped by adding 2‐M H_2_SO_4_, and the optical density at the 450‐nm wavelength (OD450) was measured using the Microplate Reader. The calculation formula of inhibition rate is as follows [17]:

$$\text{Inhibition rate}\;\left(\%\right)=\left(B_{0}-B_{i}\right)/B_{0}\times 100\%,$$

wherein *B*_*i*_ is the absorbance value (450 nm) of the experimental sample, and *B*_0_ is the absorbance value (450 nm) of the control sample, respectively.

### 2.9 Injection of Anti‐TSHR289 mAbs for In Vivo Stimulation of Thyroid Gland

Purified anti‐TSHR289 mAbs (250 μg) and sterile PBS (100 μL) were administered intraperitoneally to 6‐ to 8‐week‐old female BALB/c mice. Mice were randomly divided into four groups of 10 mice each. Mice were reared as shown previously, with all animal care and treatment procedures carried out in accordance with the Guide for the Care and Use of Laboratory Animals, which were approved by the local authorities at Lanzhou University Second Hospital, China (approval number D2025‐337). Injections were administered twice a week, once every 2 days for 3 weeks. Weight and water intake changes of mice were monitored every 2 days. Two days after the last injection, the mice were anaesthetised with isoflurane overdose, and blood and thyroid tissue were obtained. Serum total T4 levels were measured in undiluted serum (50 μL) using T4 ELISA Kit 96T (ZY‐T4‐MO, Zeye Bio‐Technology, Shanghai, China). Thyroid glands were excised for histological analysis.

### 2.10 Detection of NF‐κB Phosphorylation Levels and Chemokine Expression After Stimulation of Human Thyrocytes With Anti‐TSHR289 mAbs

#### 2.10.1 Cell Culture and In Vitro Stimulation

Nthy‐ori 3‐1 cells were disrupted with 0.25% trypsin–EDTA (25200‐056, Thermo Fisher Scientific) treatment and used between the second and the sixth passages. Cells in 60‐mm‐diameter plates were shifted to a medium containing 1% FBS for 16 h before the initiation of experimental treatments. To the confluent thyroid monolayers were added human TSH (R42K2501, Bio‐spring, Beijing, China) (1 mU/mL), 7C3 IgG (10 μg/mL), 1A4 IgG (10 μg/mL) and 22B1 IgG (10 μg/mL), and the monolayers were incubated for 60 min. PBS was added to the control plates. Nthy‐ori 3‐1 cells were pretreated with Iκκ inhibitor (Iκκ‐16, S2882, Selleckchem, Houston, USA) in 2 μM in 0.1% DMSO for 2 h and then treated with TSH (1 mU/mL) or anti‐TSHR289 mAbs (10 μg/mL) for 60 min.

#### 2.10.2 Total Protein Extraction and Western Blotting

Following the appropriate incubation time, the plates were rinsed with PBS, and the cells were lysed with RIPA Lysis Buffer (P0013B, Beyotime, Shanghai, China) supplemented with 1 mM phenylmethylsulfonyl fluoride (ST506, Beyotime). The protein concentration was determined by BCA protein Assay Kit (P0012, Beyotime). Lysates were subjected to SDS‐PAGE, and the separated proteins were transferred to a polyvinylidene difluoride membrane. After being blocked with 10% nonfat milk in 1 × TBST for 90 min, the membrane was incubated with phospho‐NF‐κB p65 Ab (ab76302, Abcam, Cambridge, UK), NF‐κB p65 Ab (80979‐1‐RR, Proteintech, Wuhan, China), phospho‐NF‐κB p100 Ab (4810, CST Inc., Danvers, MA, USA), NF‐κB p100/52 Ab(4882, CST Inc., Danvers, MA, USA), phospho‐Iκκ Ab (2697, CST Inc., Danvers, MA, USA), Iκκ Ab (YT2302, Immunoway, Suzhou, China) and CCL19 Ab (YT2766, Immunoway, Suzhou, China) overnight at 4°C, washed, and then incubated with HRP‐conjugated secondary Ab (SA00001‐2, Proteintech, Wuhan, China) for 90 min at room temperature. After washing, the bound Ab was revealed using the Immobilon Western HRP Substrate and detected with a Tanon 4600 instrument. Beta Actin Ab (20536‐1‐AP, Proteintech) was used as a control for immunoblotting.

#### 2.10.3 Total RNA Extraction and RT‐qPCR

Total RNA was extracted from cultured thyrocytes with RNeasy Plus Micro Kit (74106, Qiagen, Dusseldorf, Germany), and then reverse transcription reactions were carried out with Evo M‐MLVRT Mix Kit (AG11728, Accurate Biotechnology, Hunan, China). PCR amplifications were performed on a CFX96 Real‐Time system (Bio‐Rad, Carlsbad, CA, USA) using TB Green Fast qPCR Mix (RR430A, Takara, Kusatsu, Japan), and β‐actin was used as an internal reference. The 2^−ΔΔCt^ quantification method using β‐actin for normalisation was used to estimate the amount of target mRNA in the samples, and expression was calculated relative to the average mRNA expression levels from control samples.

#### 2.10.4 ELISA

The concentrations of CCL2, CCL19, CCL5 and IL‐1β in the cell supernatants were determined using commercial ELISA quantification kits (VAL134, R&D Systems, Minnesota, USA), (VAL163, R&D Systems), (VAL192, R&D Systems) and (VAL101, R&D Systems). The experimental procedure was performed according to the manufacturer's instructions.

### 2.11 Statistical Analysis

Statistical analysis was performed using Prism9 Software. Data are expressed as mean ± SEM. Shapiro–Wilk test and Kolmogorov–Smirnov test were used to test for normal distribution of the data. Differences between multiple groups were analysed using one‐way ANOVA or Welch ANOVA for normally distributed variables, and the Kruskal–Wallis test for nonparametric variables. *p* < 0.05 was considered significant.

## 3 Results

### 3.1 Preparation of TSHR289‐HIS Fusion Protein

**FIGURE 1:**
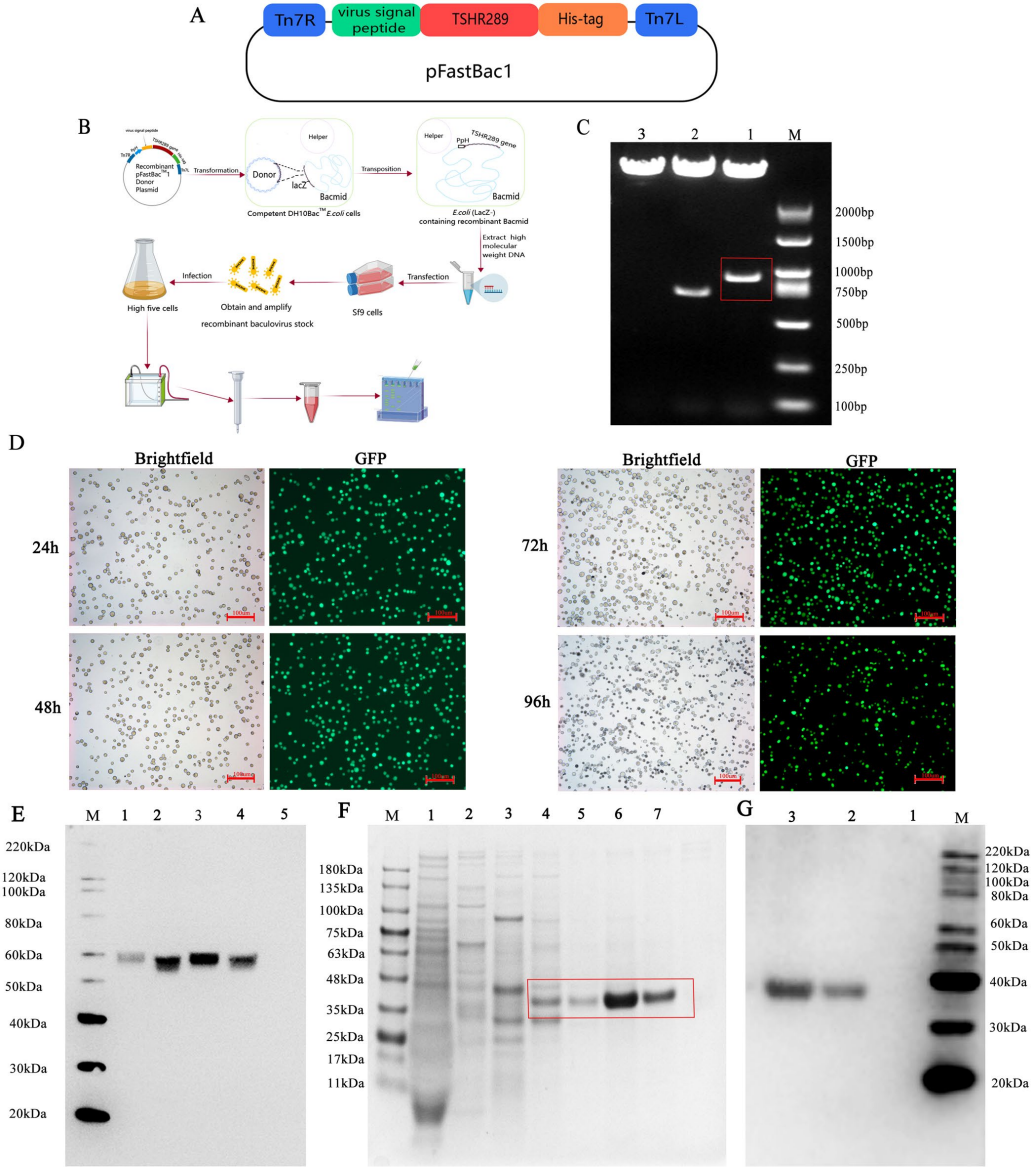
Preparation of the TSHR289‐HIS fusion protein. (A) TSHR289 gene with viral signalling peptide and HIS tag was recombined into plasmid pFastBac1 to construct pFastBac1‐TSHR289 recombinant plasmid. Different colours represent different protein‐expressing elements in the vector. (B) TSHR289‐HIS fusion protein preparation procedure. (C) Recombinant plasmids were identified by double digestion with *BamH I*/*EcoR I*. Lanes 1, 2 and 3 are double‐enzymatically digested PfastBac1‐hTHSR289, PfastBac1‐GFP and PfastBac1 plasmids, respectively. The red box represents 885 bp of the TSHR289 gene. The bands appearing at the top of lans 1, 2 and 3 are pfastbac1 vector plasmids. (D) Fluorescence microscopy of the infection efficiency of GFP‐containing baculoviruses infecting High Five cells at different time periods (100×). (E) Western blot was used to detect the expression level of TSHR289 fusion protein in cell culture supernatants at different times. Lanes 1–4 were cell culture supernatants (molecular weight 55 kDa) at 24, 48, 72 and 96 h, respectively, and lane 5 was virus‐uninfected cell culture supernatants. (F) 4%–15% SDS‐PAGE of the TSHR289 fusion protein purified by nickel column. Lanes 1–7 were samples performed by using 10, 40, 100, 100, 250, 250 and250 imidazole eluate, respectively. The red box represents the bands of deglycosylated TSHR289 protein. (G) Western blot was used to detect 250 mM imidazole eluted deglycosylated TSHR289 protein (molecular weight 35 kDa). Lane 1 was virus‐uninfected cell culture supernatants. Lanes 2, 3 were 250 mM imidazole eluted TSHR289 protein. (A, B) was created with MedPeer (medpeer.cn).

The TSHR gene with HIS‐tag was inserted into a pFastBac1 plasmid to construct a recombinant plasmid pFastBac1‐TSHR 289 (Figure 1A), and the procedures of preparation are shown in (Figure 1B). Double digestion showed destination bands with molecular weights consistent with expected sizes (Figure 1C). The predicted amino acid (aa) sequence of the TSHR289‐HIS fusion protein expressed by the recombinant TSHR289 gene, which contains a total of 294 aa, including 20 aa virus signal peptide, 268 aa of TSHR and 6 histidines (6 × HIS tags). Finally, termination codons were introduced. Recombinant baculoviruses containing TSHR289 and containing GFP plasmid infect High five cells, respectively, and Western blot was used to measure the expression of TSHR289 protein in cell culture supernatants. Fluorescence microscopy revealed stronger fluorescence expression in the cell after 48 and 72 h of infection (Figure 1D). Western blot showed that a higher content band appeared at about 55 kDa after 72 h infection at 28°C, compared with virus‐uninfected cell culture supernatants (Figure 1E). After deglycosylation, the fusion protein of TSHR289 was purified by nickel column. SDS‐PAGE showed that there was a band of TSHR289‐HIS fusion protein with some unspecific bands after purification with 100 mM imidazole elution by using a nickel column affinity layer (Figure 1F). With the 250 mM imidazole, we obtained a purer TSHR289‐HIS fusion protein (Figure 1F,G). The predicted MW of the glycosylated TSHR289 protein was about 55 kDa, and the deglycosylated TSHR289 protein was 35 kDa [18, 19], which was consistent with previous reports, indicating the success of the expression of the TSHR289‐HIS fusion protein.

### 3.2 Preparation of Anti‐TSHR289 Protein mAbs

**FIGURE 2:**
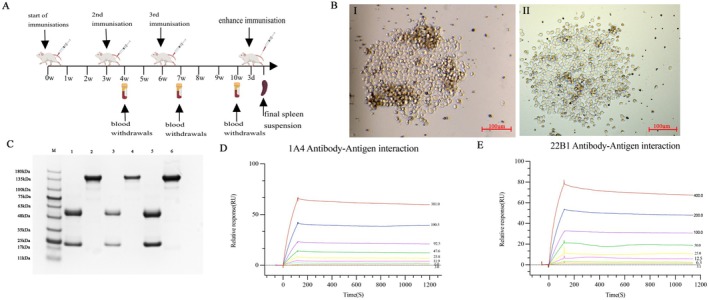
(A) Animal study schema. (B) Ⅰ and Ⅱ were hybridoma cells from day 7 after fusion of SP2/0 with splenocytes from immunized BABL/c mice (100×). (C) Reduce SDS‐PAGE analysis and non‐reduced SDS‐PAGE analysis of Anti‐TSHR289 mAbs purified by Protein G. Lanes 1, 2 were reduced and non‐reduced 7C3, respectively; lanes 3, 4 were reduced and non‐reduced 1A4, respectively; lanes 5, 6 were reduced and non‐reduced 22B1, respectively. M was Protein Marker. (D, E) Affinity measurement of the 1A4 and 22B1. Running configuration: immobilisation; ligand: TSHR 289. Association and dissociation for 1A4: flow rate, 30 μL/min; sample concentrations, 3.0, 6.0, 11.9, 23.8, 47.6, 92.5, 190.5, 381.0 nM. Association and dissociation for 22B1: flow rate, 30μL/min; sample concentrations, 3.1, 6.3, 12.5, 25, 50, 100, 200, 400 nM. (A) was created with MedPeer (medpeer.cn).

The spleen cells of mice immunised with Ad‐TSHR289 (Immunization procedure is shown in Figure 2A) were fused with SP2/0 cells by the hybridoma technique, and more than 20 hybridoma cell lines were obtained. The cell colonies were large and the growth was good (Figure 2B). Hybridomas positive were cloned three times by limiting dilution to obtain monoclonal cell lines. Finally, we obtained three monoclonal cell lines named 1A4, 7C3 and 22B1. Cell supernatants were collected and intraperitoneally injected into female BALB/c mice to produce ascites, which were collected, and the antibody was purified using a protein G prepacked column. The molecular weight of the protein in the non‐reduced state was 150 kDa (Figure 2C), and the antibody in the reduced state had two bands of 25 and 50 kDa as determined by SDS‐PAGE, which were consistent with the MWs of the light and heavy chains of mouse IgG2b, indicating that the purity of the purified mAbs was high. IgG2b subtype antibodies were found by using the capture method provided by the manufacturer's protocol of an mAb Subtype Identification Kit. The results showed the antibody isotype was mouse IgG2b, kappa. Then the affinity measurement revealed that the affinity of the TSHR 289 protein to 1A4 and 22B1 was 6.333E‐10M, 1.389E‐9M, whereas non‐specific binding was found when 7C3 was detected for the binding of the TSHR 289 protein (Table 1, Figure 2D,E). This discordance may be caused by the lysine residue in the TSHR 289 protein, which would bind to the carboxyl of the chip, blocking the binding of 7C3 to TSHR289.

**TABLE 1:** Affinity measurement of TSHR289 to 1A4 and 22B1.

Ligand	Analyte	Chi^2^ (RU^2^)	ka (1/Ms)	kd (1/s)	KD (M)
TSHR289	1A4	0.324	7.756 × 10^4^	4.912 × 10^5^	6.333 × 10^10^
	22B1	0.552	6.312 × 10^4^	8.765 × 10^5^	1.389 × 10^9^

### 3.3 Characterisation of the Anti‐TSHR289 Mabs

**FIGURE 3:**
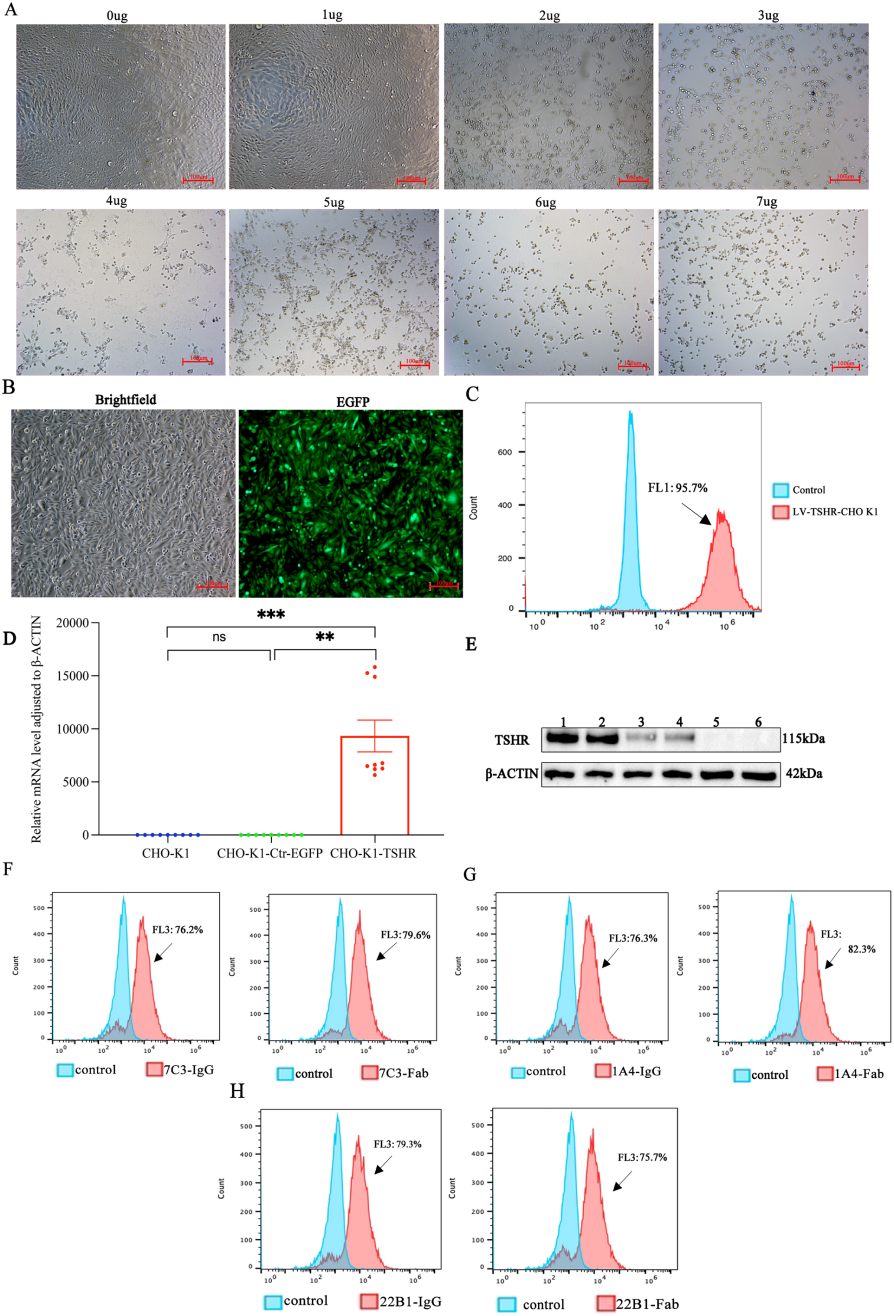
(A) Screening for the lowest concentration of puromycin to kill CHO‐k1 cells and 6 μg/mL was the optimal dose (100×). (B) Fluorescence microscopy to observe fluorescence expression in CHO‐k1 cells transfected with human TSHR full‐length (100×). (C) Flow analysis of fluorescence expression intensity in CHO‐k1 cells transfected with human TSHR full‐length versus the untransfected group. The intensity of fluorescence expression in the transfected group of cells was up to 95.7%. (D) RT‐qPCR for human TSHR gene expression in CHO‐k1 cells transfected with human TSHR full‐length, CHO‐k1 cells transfected with control EGFP and CHO‐k1 cells. The abundance of the human TSHR gene was significantly upregulated in CHO‐k1 cells transfected with human TSHR full‐length compared to controls, **p < 0.01; ***p = 0.0001. (E) Western blot was used to detect human TSHR protein expression in the transfected and untransfected groups. Lanes 1, 2 were puromycin‐screened transfected human TSHR full‐length CHO‐k1 cells; lanes 3, 4 were CHO‐k1 cells after transfection of human TSHR full‐length 48 h; lane 5 was untransfected cells; Lane6 was CHO‐k1 cells transfected with control EGFP. (F–H) Flow cytometry detected the ability of IgG and Fab of 7C3, 1A4 and 22B1, respectively, to bind to stably transfected CHO‐k1 cells with human TSHR full‐length.

To explore the ability of TSHR289 mAbs to recognise natural TSHR along with its activity, we constructed CHO cells stably transfected with TSHR full‐length using lentiviral transfection. After successful lentiviral transfection, 6ug/mL of puromycin was used to pressurize and screen stably transfected TSHR full‐length CHO‐k1 cells (Figure 3A). Fluorescence imaging and flow cytometry analysis showed strong fluorescence expression in CHO cells transfected with TSHR full‐length compared with controls (CHO‐k1 and CHO‐k1‐Ctr‐EGFP) (Figure 3B,C). Subsequently, we investigated the expression of TSHR mRNA and protein levels in CHO‐k1 cells transfected with TSHR full‐length compared to controls. The RT‐qPCR showed that TSHR relative expression levels were upregulated in CHO‐k1 cells transfected with TSHR full‐length compared to controls (Figure 3D). Western blot showed that CHO‐k1 cells transfected with human TSHR full‐length expressed TSHR protein with a molecular weight of 115 kDa [19] (Figure 3E). As shown in Figure 3F–H, flow cytometry revealed that the IgG and Fab segments of 1A4, 7C3 and 22B1 strongly stained CHO‐k1 cells transfected with TSHR full‐length, confirming their ability to bind full‐length TSHR.

### 3.4 Thyroid‐Stimulating Activity of the mAbs

**FIGURE 4:**
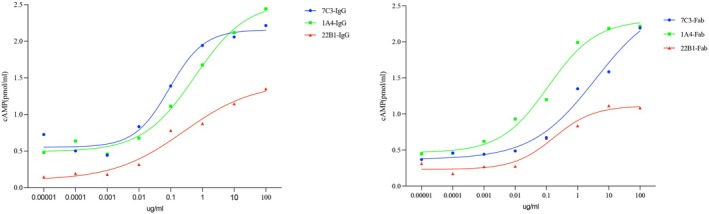
Dose–response curves of 1A3, 7C3 and 22B1 IgG and Fabs assessed by bioassay in CHO‐k1 cells transfected with TSHR full‐length. IgG and Fab of all mAbs were assayed for thyroid‐stimulating activity at different dilutions of concentration. The stimulatory activity was assayed at concentrations of 0.00001, 0.0001, 0.001, 0.01, 0.1, 1, 10 and 100 μg/mL, respectively.

As shown in Figure 4, all three mAbs stimulated cAMP production in CHO‐k1 cells transfected with the TSHR full‐length. We observed that cAMP production increased as the concentration of mAbs stimulating TSHR increased. The dose–response curve showed sigmoid curves, which were especially typical of 7C3 IgG stimulation (Figure 4). All three mAbs exhibited different thyroid‐stimulating activities. At low concentrations of mAbs (≤ 10 ng/mL), 7C3 and 1A4 stimulated higher cAMP production than 22B1. As the concentration was increased to the maximum dose of 100 μg/mL, the stimulatory activity of 22B1 was still lower than that of the other two mAbs, which was only 1.35 pmol/mL. In addition, we observed that the Fab of 7C3, 1A4 and 22B1 also exhibited thyroid‐stimulating activity similar to that of intact IgG.

### 3.5 Competition Studies With 1A3, 7C3 and 22B1

**FIGURE 5:**
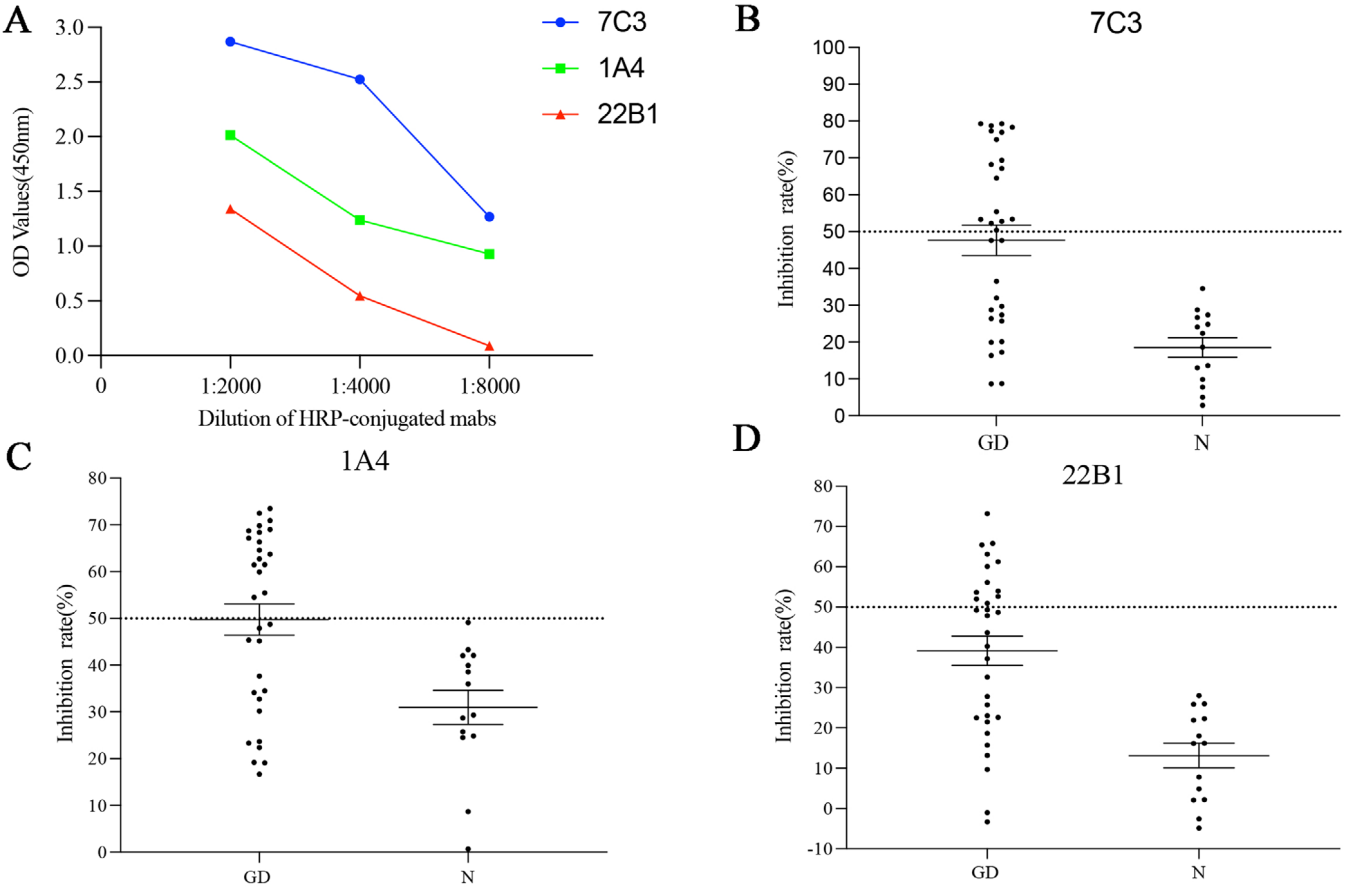
(A) ELISA examined the ability of HRP‐conjugated mAbs at different ratios of dilution to bind to TSHR; (B–D) Serum TSHR autoantibodies from different GD patients competitively inhibited the binding of each of the 3 mAbs to TSHR.

The specificity of anti‐TSHR289 mAbs was confirmed by the results of the competitive binding assay, which showed that TRAb in the sera of GD patients could competitively inhibited the binding of TSHR289 mAbs to TSHR. The ability of the anti‐TSHR289 mAbs to effectively bind TSHR was analysed in direct ELISA experiments. 8000‐, 4000‐ and 2000‐fold dilutions of HRP‐conjugated 7C3, 1A4 and 22B1 were selected as the optimal dilution concentrations for competitive ELISA, respectively (Figure 5A). We found that autoantibodies to TSHR in the sera of GD patients competitively inhibited the binding of 7C3, 1A4 and 22B1 to TSHR coated on ELISA plates with inhibition rates of 53%, 53% and 37.5%, respectively (Figure 5B–D). Serum from 14 normal individuals without any disease record was used as a control.

### 3.6 Effects of 1A4, 7C3 and 22B1 on Thyroid Function In Vivo

**FIGURE 6:**
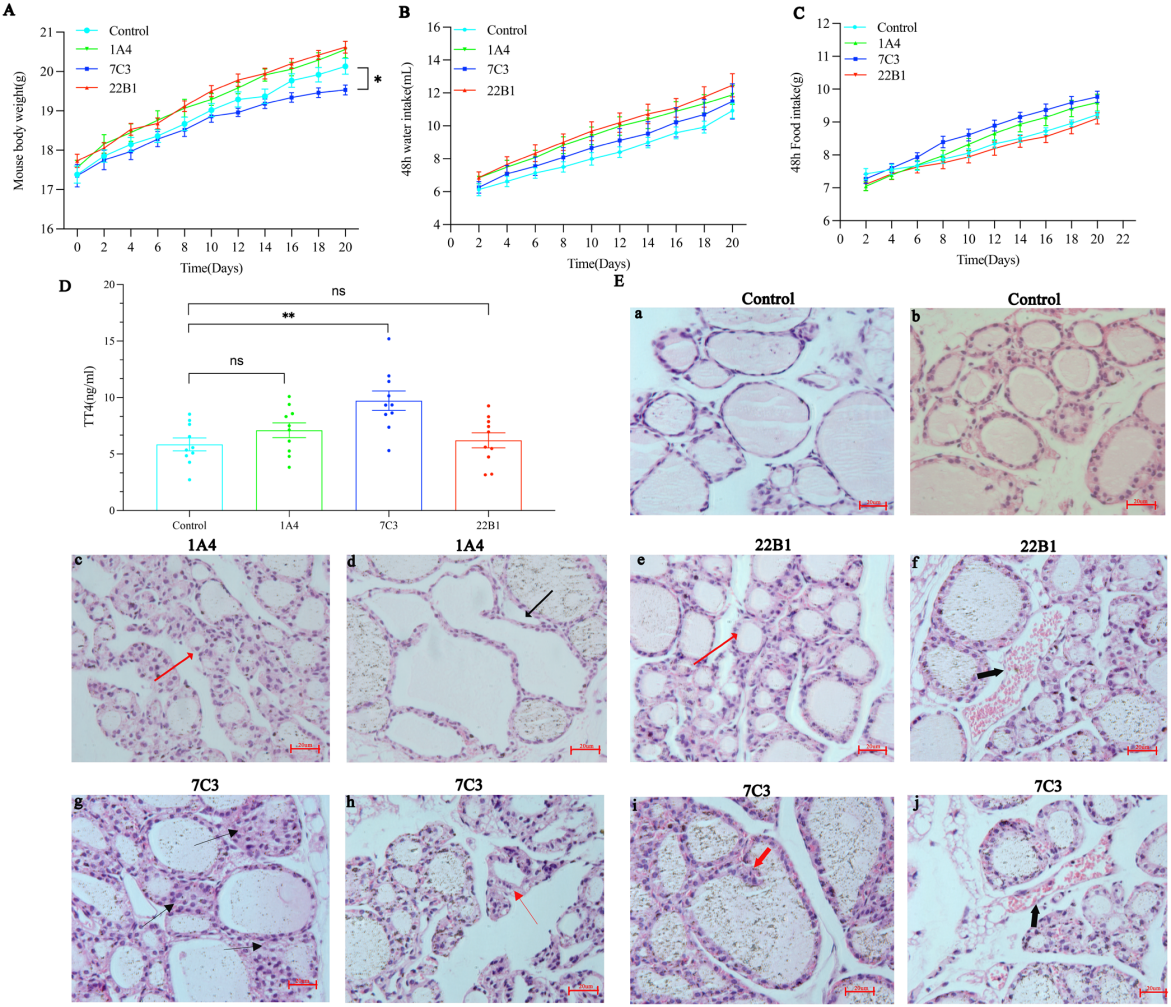
(A) Body weight change curves of PBS, 1A4, 22B1 and 7C3 injected mice, *p < 0.05; (B) Curves of changes in water intake of mice at 48 h after injection of PBS, 1A4, 22B1 and 7C3 into mice; (C) Curves of changes in food intake of mice at 48 h after injection of PBS, 1A4, 22B1 and 7C3 into mice; (D) Serum TT4 assay in 1A4, 7C3, 22B1‐injected female BALB/c mice, **p < 0.01; (E) HE sections of thyroid glands of control and mAbs‐injected BALB/c female mice: (a, b) HE sections of thyroid glands from PBS‐injected BALB/c female mice (400×). (c, d) HE sections of thyroid glands from 1A4‐injected BALB/c female mice (400×): (c) mild hyperplasia, (d) follicular expansion; (e, f) HE sections of thyroid glands from 22B1‐injected BALB/c female mice (400×): (e) mild hyperplasia, (f) interstitial vasodilatation and congestion; (g–j) HE sections of thyroid glands from 7C3‐injected BALB/c female mice (400×) :(g) loss of thyroid colloid formation with multilayering and luminal collapse, (h) columnar hyperplasia, (i) papillary hyperplasia, (j) interstitial vasodilatation and congestion.

We examined whether the in vitro effect of 1A4, 7C3 and 22B1 would be reflected in vivo. The body weight of the 7C3‐injected mice increased slowly compared with that of the control group, especially 1 day before execution (*p* < 0.05, Figure 6A), and no significant changes were observed in the remaining two groups. Water intake and food intake at 48 h did not differ between mAbs‐injected mice and control mice, and both showed a steady increase in water intake and food intake (Figure 6B,C). Serum total T4 levels after 1A4 injection were 7.1 ± 0.7 ng/mL (MEAN ± SEM; *n* = 10) (Figure 6D). Serum total T4 levels after 22B1 injection were 6.2 ± 0.7 ng/mL. Serum T4 was significantly elevated in the mice receiving 7C3 (9.7 ± 0.9 ng/mL) than in the animals receiving PBS (5.9 ± 0.6 ng/mL) (*p* < 0.05). Histological analysis of the thyroid glands of mice injected with 1A4, 22B1 and 7C3 showed alterations in follicular epithelial cells and follicles compared to control mice (Figure 6E). Mild hyperplasia of the thyroid follicular epithelium was observed in 1A4‐ and 22B1‐treated mice compared with controls (Figure 6E‐c,e). Thyroid follicles of 1A4‐treated mice appeared to be markedly dilated (Figure 6E‐d), and interstitial blood vessels in the 22B1 group were dilated and congested (Figure 6E‐f). Loss of thyroid colloid formation with multilayering and luminal collapse (Figure 6E‐g), columnar hyperplasia of follicular epithelial cells (Figure 6E‐h) and even papillary hyperplasia (Figure 6E‐i), and dilated and congested interstitium (Figure 6E‐j) in mice treated with 7C3.

### 3.7 mAbs Activate Canonical and Non‐Canonical NF‐κB Pathways and Induce Chemokine Expression

**FIGURE 7:**
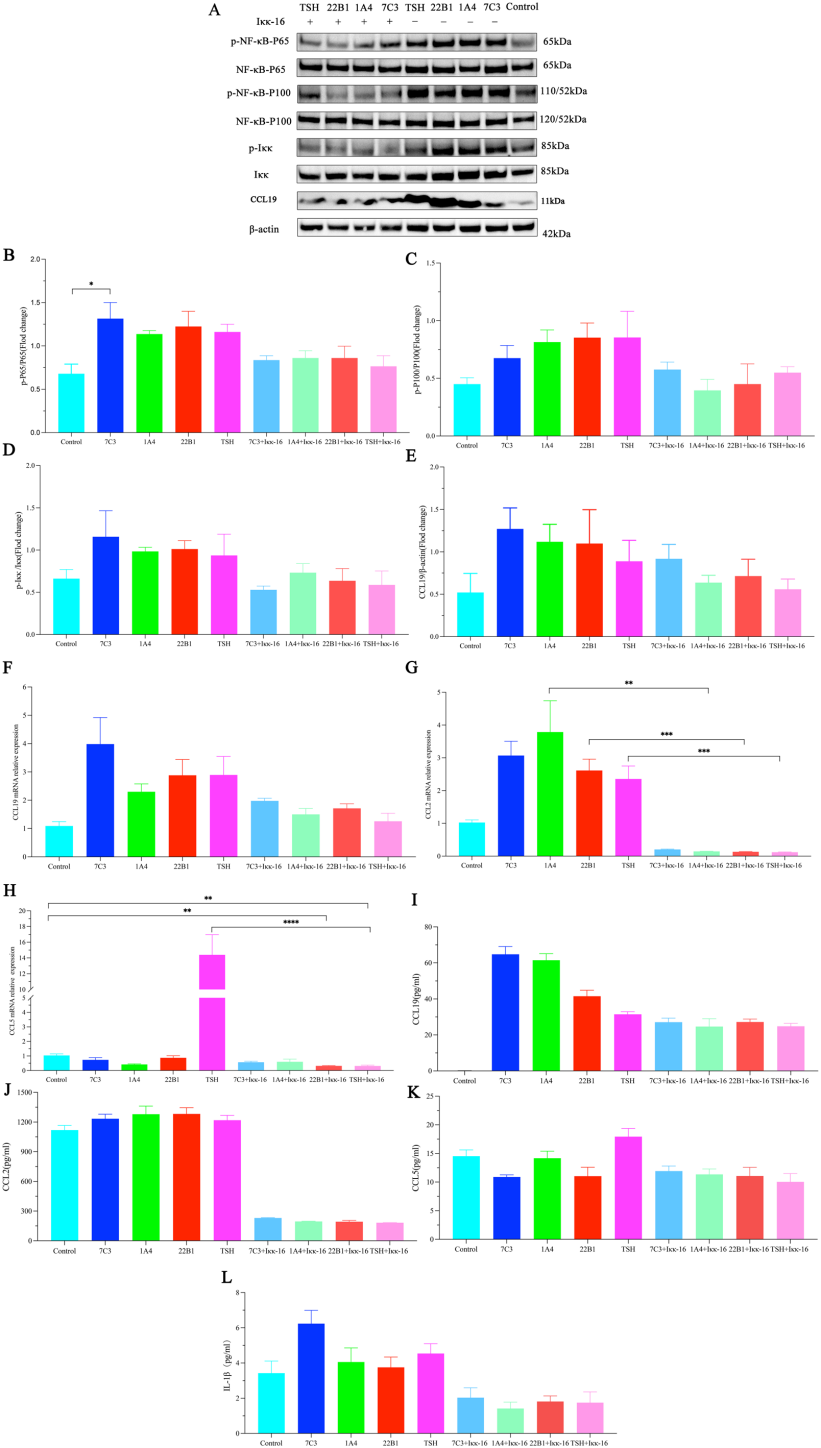
(A–E) Human thyrocytes were treated with buffer (Control), 7C3, 1A4, 22B1 and human TSH, or with Iκκ inhibitor Iκκ‐16 before treatment with the three mAbs and TSH, and the proteins were extracted for Western blotting analysis. Phosphorylated and non‐phosphorylated NF‐κB, CCL19 and β‐Actin were detected; (F–H) The levels of mRNA expression of CCL19, CCL2 and CCL5 on human thyrocytes in different treatment groups; (I–L) The levels of protein expression of CCL19, CCL2, CCL5 and IL‐1β on human thyrocytes in different treatment groups. *p < 0.05; **p < 0.01; ***p = 0.0001; ****p < 0.0001.

A number of reports have appeared recently suggesting that TSH or TSHR autoantibodies activate nuclear factor κB (NF‐κB) transcription factors in thyroid cells. NF‐κB activates the promoter regions of many chemokines and is independently implicated in the development of impaired self‐tolerance. We found that 7C3, 1A4 and 22B1 could stimulate the phosphorylation of canonical (p‐NF‐κB P65) and noncanonical NF‐κB (p‐NF‐κB P100) in thyrocytes (Figure 7A–D), and that the three mAbs acted similarly as well as mimicked the action of human TSH. 7C3, 1A4, 22B1 and TSH‐stimulated thyrocytes induced elevated expression of CCL19 and CCL2 upon activation of canonical and noncanonical NF‐κB (Figure 7A,E–G,I,J). Especially in 1A4, 22B1 and TSH‐stimulated cells, the expression of CCL2 was significantly down‐regulated after canonical and noncanonical NF‐κB was inhibited (Figure 7G). However, CCL5 expression was down‐regulated in 3 mAbs‐stimulated thyrocytes and up‐regulated in TSH‐stimulated thyrocytes (Figure 7H,K), which suggests that the anti‐TSHR antibody may exert a biological effect similar to, but not identical with TSH. When the NF‐κB signalling pathway was inhibited, we found that CCL5 expression was down‐regulated in 7C3, 22B1 and TSH‐intervened thyrocytes, and especially TSH showed significant down‐regulation. The 22B1 and TSH‐treated cells appeared significantly down‐regulated after Iκκ inhibitors intervention compared to the control. In addition, we measured the inflammatory cytokine IL‐1β associated with CCL19, CCL2 and CCL5 using ELISA. We found that both mAbs‐ and TSH‐treated cells showed elevated levels of IL‐1β, whereas cells that were pre‐intervened with the addition of an Iκκ inhibitor showed a decrease in the levels of inflammatory cytokines (Figure 7L).

## 4 Discussion

Here we report the successful cloning of three monoclonal TSHR antibodies (1A4, 7C3 and 22B1) from mice immunised with an adenoviral vector incorporating human TSHR A subunit (Ad‐TSHR A). All three mAbs recognised TSHR antigen stably transfected on CHO cells and had thyroid‐stimulating activity. Low concentrations of 7C3 and 1A4 were able to stimulate cAMP production in CHO cells transfected with human TSHR full‐length. Although 22B1 did not show the same strong stimulatory activity as the other two antibodies, it did show some stimulatory activity at the nanogram level. These thyroid‐stimulating characteristics of mAbs are similar to those of TRAb found in sera from GD [19, 20, 21]. Serum TRAb from GD patients competitively inhibits the binding of 1A4, 7C3 and 22B1 to TSHR. This suggests that the mAbs reported in this study show the characteristics of TSHR autoantibodies in the sera of GD patients, which is also consistent with the report of Sanders [22].

We observed a slow increase in body weight in the 7C3‐injected mice compared to the control group. In particular, a significant difference in body weight occurred between 7C3‐injected mice and control mice before the mice were executed. In addition, we observed that serum TT4 levels in mice injected with purified 1A4 and 22B1 mAbs did not show significant differences from controls, whereas serum TT4 was elevated in 7C3‐injected mice. Interestingly, morphological changes were seen in the thyroid gland of all three mAbs‐injected mice. All three mAbs‐injected mice showed proliferative changes in thyroid follicular epithelial cells. In 7C3‐injected mice, there was a loss of thyroid colloid formation with multilayering and luminal collapse, which is consistent with the pathological changes in the mouse thyroid induced by a potent thyroid‐stimulating mAbs [23], as reported previously. Whereas our mAbs seem to be more potent in its stimulatory effect in vivo, since we observed even pathological changes of columnar and papillary hyperplasia in the thyroid gland of 7C3‐injected mice.

Previous studies have reported that stimulatory TSHR antibodies and TSH activate the canonical NF‐κB pathway on thyrocytes [8, 24, 25]. This is confirmed and complemented by our study, in which we found that TSH and stimulatory TSHR antibodies activate both canonical and noncanonical NF‐κB pathways. The NF‐κB family of dimeric transcription factors remains dormant in unstimulated cells. p65 is the key heterodimeric transcription factor of the canonical NF‐κB pathway, whereas nonclassical NF‐κB transcription factors are only stimulated; NF‐κB activates Iκκ, which phosphorylates inactivated p100 to produce p52 [26]. The canonical NF‐κB pathway primarily encodes for chemokines responsible for the recruitment of inflammatory and phagocytes of the innate immune system. The noncanonical NF‐κB pathway mainly upregulates various proteins required for B cell viability and function, proper antigen presentation and adaptive immunity [27].

Although previous studies have reported the involvement of CCL2 and CCL5 in the pathogenetic progression of GD [14, 15], few CCL2 and TRAb studies have been reported and there is controversy over the study of CCL5 and TRAb. It has been shown that GD‐IgG induces CCL5 expression in human thyroid cells in a time‐dependent manner [28], however, another study concluded that CCL5 is negatively correlated with TRAb [16]. Our study confirms that TSHR autoantibody induces CCL2 and CCL19 expression by activating canonical and non‐canonical NF‐κB pathways. In addition, TSHR autoantibody induced downregulation of CCL5 expression. Interestingly, the expression of chemokines induced in mAbs—and TSH‐stimulated cells was not entirely consistent. CCL2 and CCL19 expression was upregulated in mAbs—and TSH‐treated cells, but CCL5 expression was downregulated in mAbs‐treated cells and significantly upregulated in TSH‐treated cells. However, when the NF‐κB pathway was inhibited, most of these chemokines showed down‐regulated expression in mAbs—and TSH‐stimulated cells. This may also confirm that TSHR antibodies may exert biological effects similar to, but not identical with, those of TSH [29]. Although they may induce chemokines to be expressed differently, they all regulate chemokine expression through activation of canonical and non‐canonical NF‐κB pathways. Indeed, previous studies have demonstrated that the NF‐κB signalling pathway is activated during oxidative stress and viral infection, which induces the expression of CCL2 [30, 31].

It has been reported that in autoimmune diseases, the formation of an inflammatory microenvironment induces the production of CCL2 in response to inflammatory cytokines such as IL‐1β, which recruits and expands macrophage aggregation at the site of inflammation [32, 33]. CCL19 synergistically stimulates T‐cell proliferation and Th1 polarisation with IL‐1β [34], and CCL5 is also thought to be closely related to IL‐1β [35]. In this study, we found that IL‐1β was elevated in cell supernatants after stimulation of thyroid cells with mAbs and thyroid‐stimulating hormones. After early intervention of cells with the addition of Iκκ inhibitors, IL‐1β levels were reduced in mAbs and TSH‐treated samples. NF‐κB can regulate pro‐inflammatory cytokine expression in adaptive immunity. NF‐κB rapidly activates and induces the synthesis of various pro‐inflammatory cytokines, such as IL‐1β [36], during immune cell damage. Our study also confirms this view.

**FIGURE 8:**
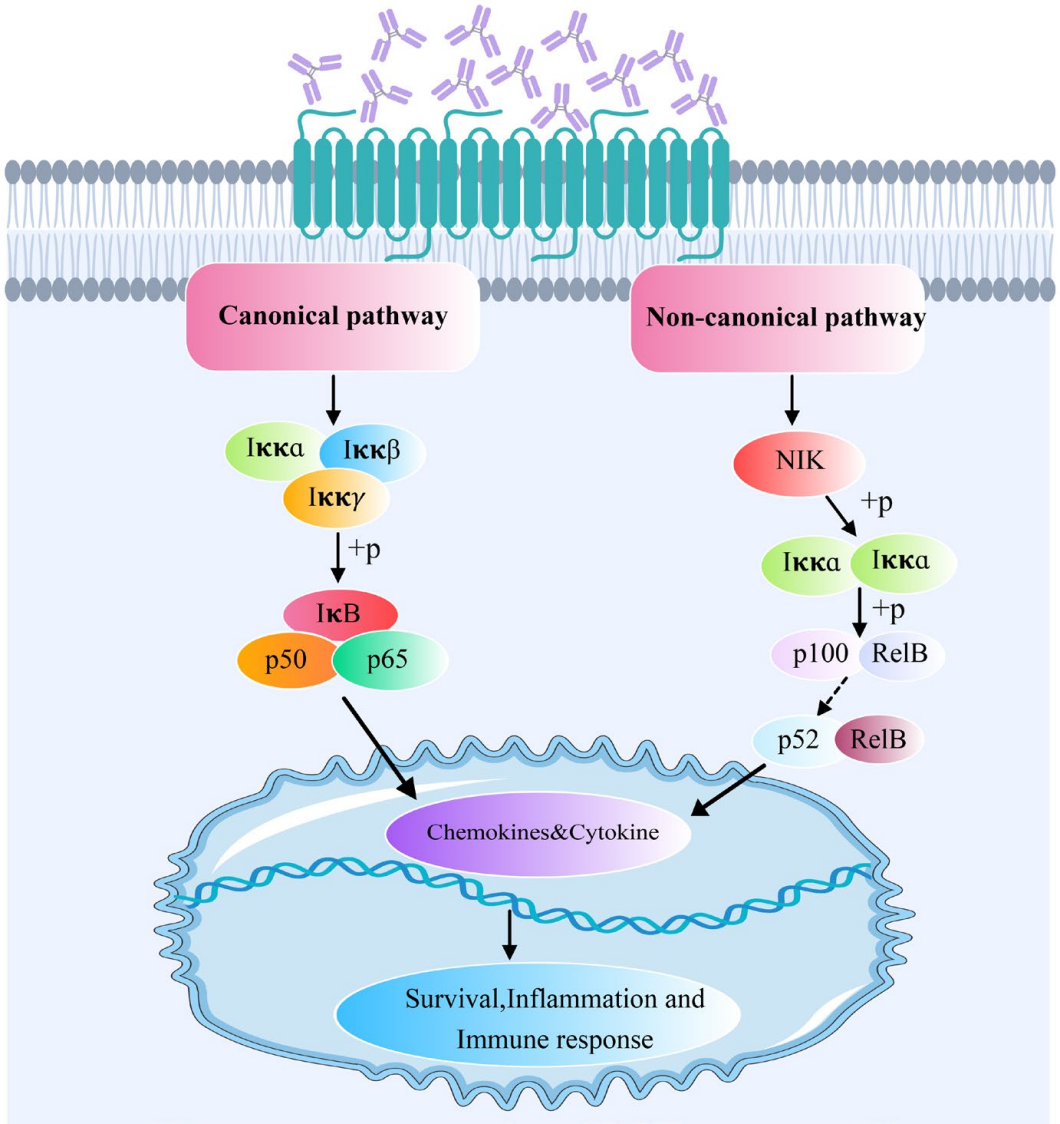
In the canonical NF‐κB signalling pathway, the NEMO complex composed of Iκκɑ/ Iκκβ /Iκκ phosphorylates IκB, which leads to its ubiquitination and subsequent proteasomal degradation. P65/p50 transcription factors are released to activate the transcription of target genes. In the non‐canonical NF‐κB signalling pathway, NIK is activated and phosphorylates Iκκ, p100 is phosphorylated and processed into p52, which binds RelB transcription factors and translocates into the nucleus to activate the expression of specific genes. Canonical and non‐canonical NF‐κB signalling pathways together mediate cellular inflammation and immune responses. Created with MedPeer (medpeer.cn).

The development of thyroid‐stimulating mabs such as 1A4, 7C3 and 22B1 adds to the theoretical basis and a favourable tool for molecularly dissecting TSHR and epitopes associated with autoimmune thyroid diseases. This will help to refine the intracellular signalling patterns on TSHR associated with thyroid stimulation. The generation of anti‐unique antibodies against 1A4, 7C3 and 22B1 for the development of CART therapies that recognise the presence of anti‐TSHR antibodies in patients with GD may enable future studies to link their response to treatment, thereby tailoring treatment to individual patients and reducing the risk of relapse. Stimulatory TSHR antibodies induce differential expression of CCL2, CCL19 and CCL5 through canonical and non‐canonical NF‐κB pathways, suggesting that these chemokines may be involved in the recruitment process of thyroid lymphocytes, thus providing important insights into the pathogenesis of GD (Figure 8). Our data deepen our understanding of the pathogenesis of GD and provide new strategies for the treatment of hyperthyroidism.

## Author Contributions

**Yang Yang:** investigation (equal), methodology (equal), writing – original draft (equal). **Chen Hui:** funding acquisition (equal), writing – review and editing (equal).

## Conflicts of Interest

The authors declare no conflicts of interest, This study has been applied for Chinese national invention patent.

## Data Availability

The authors have nothing to report.
